# Wisdom from the past

**Published:** 1970-07

**Authors:** 


### Wisdom from the past

(Extracts from This Journal, 1895 and 1920)

### SEVENTY-FIVE YEARS AGO

**From "Notes on Egypt as a Health Resort" by P. Watson Williams, M.D., Assistant Physician to the Bristol Royal Infirmary.**

"The Northern African seaboard has long been recognised as a region which presents many and great attractions as a winter resort for sufferers from various affections of the chest, and which is, at the same time, easily accessible to Europeans. Certain regions, more especially Algiers, have long successfully competed with the Riviera for the patronage of French and English patients who are compelled to exchange the cold and damp of their own less favoured winter for a more genial climate.

Egypt is a land of all-engrossing interest, both in its historic remains and in the varied phases of modern life that are ever before the traveller's eye; and as it is so rapidly becoming one of the most popular winter resorts, I have endeavoured, in the narrow limits of the present article, to indicate the various routes by which it may be reached, the character of the climate, cost of living, and the various affections for which it is most suitable, together with other practical points which may aid the practitioner in choosing a health-resort suited to the condition of health and the means of his patients.

For those who greatly dislike the sea voyage, Alexandria is the best port of entry, as it is only six days journey from London (by Paris, thence to Marseilles, fourteen hours, Messagerie steamboat to Alexandria, three days); or, going overland to Brindisi, the P. and O. boat can be taken for Alexandria. After a night's rest, one proceeds to Cairo by the morning express, a three hours run. Another route has recently been provided by the P. and O. Company by boat from Venice to Port Said, or by the Orient line from Naples to Port Said, five days. The long sea route from London or Plymouth to Port Said is often to be preferred for those who are good sailors—ten days at sea. One can sleep at Port Said if the boat arrives at night; otherwise it is better to go on to Ismailia, four hours by rail, where there is an excellent inn, now taken over as a succursal to Shepheard's Hotel (Cairo); or, if the patient is strong enough, the remainder of the journey by rail to Cairo may be accomplished in another four hours.

In Egypt everyone is, or should be, a "Cook's tourist", there is then no fear of being pestered by the Clamorous crowd of native porters. The ubiquitous "Cook" boards the steamers, and one has only to hand the luggage checks to the agent, and mention the name of the hotel one is going to, without giving it another thought. If one cares to convert a peaceful journey into one long and incessant worry by officious natives who understand nothing but Arabic, unless it is a very big D—, one has only to try a journey without "Cook".

Cairo has about 430,000 inhabitants, and on leaving the new and handsome station to be driven to one of the many large first-class hotels, we soon realise that, with all the brilliant pictures of Oriental life which surrounds us on every side, we have landed in a city where all the comfort and luxury of home life obtain. At almost all the first-class hotels in Cairo, Mena House, and at Luxor and Assouan, board and lodging come to about sixteen shillings a day, or less (fourteen shillings and sixpence) by the month, this including everything except wine, washing, and light. At Helouan Hotel the charge is 8/6 to 10/6 a day, while at the very comfortable *Pension* Antonio for those who are not strictly invalids the inclusive charge is only seven shillings a day".

(Vol. 13, pp. 263-265; 1895)

### MALE NURSES

"It must have been in the experience of most medical men that there is considerable difficulty in obtaining male nurses to attend on those who, from their peculiar ailments, rather resent the presence and manipulations of a woman. We have much pleasure in calling attention to the existence of an institution where not only male nurses can be obtained, but where the sobriety of the attendant is guaranteed, a by no means unimportant matter when dealing with the male sex. The Male Nurses (Temperance) Co-operation has been in existence for more than a year, and from the need there is for trustworthy sober men for nursing, we believe that only a more thorough knowledge that there is such an institution is required for its continued success. The secretary's address is 8 Great Marylebone Street, London, W."

(Vol. 13, p. 234; 1895)

### FIFTY YEARS AGO

**From "Famine-Dropsy as a Food-Deficiency Disease" by J. A. Nixon, C.M.G., M.D., F.R.C.P.**

"Under the names of War Oedema and Hunger Oedema an old form of dropsy was described during the Great War as a new disease. Dropsy had been recognised as the result of under-feeding and famine since the time when the soldiers of the French Army before Naples in 1528 had crawled about with pallid visages, swelled legs, and bloated bellies. It had been recorded in besieged towns, in ships making long cruises and in famine-stricken countries. Great confusion had existed until recent times between famine dropsy, scurvy and beriberi. Cornish described the condition with great accuracy as occurring in the Madras jails in 1864, and again in the Indian famine of 1877-8.

During 1915 outbreaks occurred in German prison camps in association with various epidemic diseases, such as relapsing fever, dysentery and typhus. At the same time it was observed among the famine-stricken populations in Galicia and Poland. From a recently-published report it is evident that the earliest appearance of famine-dropsy during the War was at Lille in October, 1914. The unhappy town was in German occupation, and the inhabitants were suddenly stripped of everything, so that many of them had nothing to eat except potatoes. This diet produced many cases of a general anasarca unaccompanied by albuminuria. My own experience of the disease was derived from seeing a number of slight and a few severe cases amongst released prisoners of war who were making their way back to France immediately after the Armistice, or who were found in the hospitals of the re-occupied portions of France and Belgium during the advance of the Fourth Army."

(Vol. 37, pp. 137-138; 1920)

**From "Poisoning by 'Mustard' Gas" by**

**E. H. E. Stack, F.R.C.S., Capt., R.A.M.C. (T.F.)**

**Carey F. Coombs, M.D., F.R.C.P., Capt., R.A.M.C. (T.F.)**

**and R. Rolfe, B.A., M.B., B.Ch.**

"On the night of July 25th, 1917, 40 patients were admitted to No. 56 General Hospital suffering from gas poisoning of a new type, and three nights later 27 more were admitted. Owing to the prevalence of eye symptoms among them they were principally under the care of one of us (E.H.E.S.), while the other (C.F.C.) was told off by Col. J. Paul Bush, C.M.G., O.C. No. 56 General Hospital, to collect general data. The earlier part of this paper is taken from our report to Col. Bush, presented on Aug. 3rd.

Of the first batch of 40 men all had been gassed at Armentieres between the 21st and 24th July, the source of the gas being enemy shells in every case. The second batch all came from Ypres; the majority had been gassed between 12th and 16th July.

Taking the 67 men together, we found that 23 were gassed only while in the open, the same number only while in cellars or dug-outs, 4 were gassed both in the open and in dug-outs, while the remainder (7) were not sure about the source. Gas helmets were used in 53 instances, the remainder consisting of men gassed in their sleep, and others who stated that they received no orders to put on helmets. Most of those who put on helmets thought that they failed to protect because they were taken off too soon under the mistaken impression that the gas had disappeared.

Of the men gassed in dug-outs several stated that there was no adequate protection against gas. In others the shell either entered the dug-out or destroyed its protection.

An extraordinary series of similes was offered when the men were asked what the gas smelt like, the general impression being that it was a bad smell, and the most popular comparisons being to some kind of vegetable decay.

The symptoms produced by the gas (which are now well known) were those of irritation of eyes, nose and stomach. In all but two cases there was eye irritation with lachrymation as a first symptom. Unlike the effects of lachrymatory gas, however, these did not follow gassing until after an appreciable interval, varying from 1 to 48 hours, and averaging nearly 7½ hours. Symptoms of nasal irritation occurred in 62 cases and headache in 56. Fifty-seven of the men vomited or retched, the average interval between gassing and the onset of this symptom being about 6½ hours. The throat became sore in 59 cases, the earliest symptom being felt on an average about 16 hours after gassing. In addition to these there were skin symptoms, to be referred to presently."

(Vol. 37, pp. 152-153; 1920)

